# V0162 a new long-acting bronchodilator for treatment of chronic obstructive lung diseases: preclinical and clinical results

**DOI:** 10.1186/s12931-015-0227-1

**Published:** 2015-06-08

**Authors:** Philippe Devillier, Eric Garrigue, Guillaume D’Auzers, Nicolas Monjotin, Thomas Similowski, Thierry Clerc

**Affiliations:** UPRES EA 220, Hôpital Foch, Université de Versailles Saint Quentin, 11 rue Guillaume Lenoir, Suresnes, 92150 France; Centre de Recherche et de Développement Pierre Fabre Toulouse, 3 Avenue Hubert Curien BP 13562, 31035 Toulouse, France; Institut de Recherche Pierre Fabre, Service de Pharmacologie, CEPC Bel Air de Campans, Castres Cedex, 81106 France; AP-HP, Groupe Hospitalier Pitié-Salpêtrière Charles Foix, Service de Pneumologie et Réanimation Médicale (Département “R3S”), 47-83 Bd de l’Hôpital, F-75013 Paris, France; Sorbonne Universités, UPMC Paris 06, UMR_S 1158 “Neurophysiologie Respiratoire Expérimentale et Clinique”, F-75005 Paris, France; INSERM, UMR_S 1158 “Neurophysiologie Respiratoire Expérimentale et Clinique”, F-75005 Paris, France

**Keywords:** V0162, Chronic obstructive pulmonary disease, Allergic asthma, Bronchodilation, Long action, Anti-inflammatory properties, Phase I/II clinical study

## Abstract

**Background:**

Long acting bronchodilators are the standard of care in the management of chronic obstructive pulmonary disease (COPD). The aim of this study was to investigate the efficacy and safety of V0162, a novel anticholinergic agent with bronchodilator properties, in preclinical models and in patients with COPD.

**Methods:**

Guinea pigs were used to evaluate the impact of V0162 on the acetylcholine or histamine-induced bronchoconstriction. V0162 was also investigated in an allergic asthma model on ovalbumin-sensitized guinea pig. For clinical investigations, healthy volunteers were included in a dose-escalation, randomized, placebo-controlled phase I study to determine the maximal tolerated dose, followed by a randomized, placebo-controlled, cross-over phase II study in patients with COPD. V0162 was given via inhalation route. The objectives of the phase I/II study were to assess the safety and efficacy of V0162, in terms of bronchodilation and reduction in hyperinflation.

**Results:**

Preclinical results showed that V0162 was able to prevent bronchoconstriction induced either by acetylcholine or histamine. V0162 reversed the bronchoconstriction and airway inflammation caused by ovalbumin challenge in sensitized guinea pigs. In the healthy volunteers study, 88 subjects were enrolled: 66 received V0162 and 22 received placebo. No particular safety concerns were raised. The maximal tolerated dose was not reached and the dose escalation was stopped at 2400 μg. A total of 20 patients with COPD were then enrolled. All patients received a single-dose of V0162 1600 μg and of placebo in two alternating periods. In COPD patients, V0162 demonstrated a significant increase in FEV_1_ compared with placebo (148 ± 137 ml vs. 36 ± 151 ml, *p* = 0.003). This bronchodilatory effect was corroborated by a reduction in hyperinflation. There was a trend toward dyspnea relief (change in visual analog scale at 22 h, −15.1 ± 26.0 mm vs.- 5.3 ± 28.8 mm with placebo, *p* = 0.054). No serious adverse events (AEs) were reported. Most common AEs were productive and non-productive cough, dyspnea and pruritus.

**Conclusions:**

V0162 improved pulmonary function and tended to improve dyspnea in patients with COPD over more than 24 h. The slight plasmatic exposure observed might support the good safety profile.

**Trial registration:**

ClinicalTrials.gov identifier: NCT01348555

## Background

Chronic obstructive pulmonary disease (COPD) is a progressive debilitating condition and a leading cause of morbidity and mortality [1]. This common preventable and treatable disease is characterized by a persistent airflow limitation that is usually progressive and associated with an enhanced chronic inflammatory response in the airways and the lung to noxious particles or gases. Symptoms include dyspnea, chronic cough and sputum production. Exacerbations and comorbidities contribute to the overall severity in individual patients [2].

Inhaled bronchodilators are the cornerstone of symptomatic management of COPD [2]. According to the Global Initiative for Chronic Obstructive Lung Disease (GOLD), long-acting β_2_-agonists and anticholinergics are preferred over short-acting formulations in patients with persistent dyspnea [2]. Currently most available inhaled long-acting bronchodilators provide between 12- and 24-h duration of action. Of these, tiotropium was the first available long-acting anticholinergic agent providing 24 h of bronchodilation [3–7]. Tiotropium is associated with sustained reduction in lung hyperinflation and significant improvement in exertional dyspnea [7].

In the large UPLIFT study (Understanding Potential Long-Term Impacts on Function with Tiotropium), treatment with tiotropium over 4 years was associated with improvement in lung function and quality of life, and with reduced mortality, particularly cardiovascular mortality [8–10]. Tiotropium was also associated with a decreased risk of exacerbations and related hospitalizations [9]. However, tiotropium did not significantly reduce the global rate of decline in FEV_1_, but tended to prevent the decline in post-bronchodilator FEV_1_ in patients with GOLD stage II COPD [10]. A recent meta-analysis also suggested that when compared with ipratropium bromide, tiotropium was associated with improved lung function and quality of life, and fewer CODP-related exacerbations and associated hospitalizations [11].

Novel long-acting muscarinic receptor antagonists (LAMAs) are currently in clinical development for the treatment of COPD or asthma, and some were recently approved and brought to the market for the treatment of COPD (e.g. glycopyrronium, aclidinium) [12]. Glycopyrronium and aclidinium are expected to produce similar improvement in lung function, dyspnea, quality of life and exacerbation compared to tiotropium [13–15].

V0162 inhalation powder is a new anticholinergic compound derived from mequitazine, an existing oral racemic antihistamine commercialized for over 30 years. The molecular formula for V0162 is 10-[(3R)-1-azabicyclo[2.2.2]oct-3-ylmethyl]-10H-phenothiazine and its molecular weight is 322 g/mol. V0162 is the dextrorotatory enantiomer of mequitazine. Mequitazine is an antihistaminic with one of the highest affinity for muscarinic receptors in the nanomolar range [16, 17]. V0162 is developed under a pharmaceutical formulation different than that of the parent compound and at a lower dose for inhalation. In *in vitro* metabolism studies, a marked difference was observed between the levorotary and the dextrorotatory enantiomers, assuming that these latter involve different metabolic pathways. V0162 is currently under investigation mainly for the treatment of COPD.

The present paper reports results from preclinical experiments and clinical phase I/II study assessing the efficacy and safety of V0162. The aim of this study was to assess (i) the bronchodilatory and anti-inflammatory properties of V0162 in preclinical models, (ii) its safety in healthy volunteers and, (iii) and its effect on pulmonary function and dyspnea in COPD patients with hyperinflation.

## Methods

### Preclinical experiments

#### Ethical aspects

Animals were handled and cared in accordance with the European Directive 86/609, and the protocols were carried out in compliance with French regulations and with local Ethical Committee guidelines for animal research (CEA-CEPC N°110).

### Animal model for acetylcholine- and histamine-induced bronchoconstriction

Male Guinea pigs, weighing 400–450 g and divided into groups of 7–9 animals, were anaesthetized with 60 mg/kg of pentobarbital sodium. Four fifths (4/5th) were administered intraperitonealy (i.p) and the remaining fifth subcutaneously. The jugular vein and trachea were cannulated; to suppress spontaneous respiratory movements, animals were paralyzed with intravenous administration of gallamine triethioride (10 μg/kg; 1 mL/kg). The bronchopulmonary function was measured according to Konzett & Rossler’s modified method [18]. Briefly, the animals were ventilated by means of a sinusoidal pump (Ugo Basile) at constant pressure and with a constant volume in excess (5 mL) and a frequency of 90 strokes/min. The excess of air that did not ventilate the lung was measured by a transducer. An intravenous administration of acetylcholine or histamine induced a bronchoconstriction that was measured by the increase of recorded air volume in excess; the recorded ‘so-called’ residual volume (mL) corresponded to the difference between the air pump (5 mL) and the air inspired by the animal. Ventilatory parameters were recorded continuously for 75 min (IOX2, EMKA Technologies, Paris).

Bronchoconstriction was induced by intravenous (i.v.) acetylcholine (20 μg/kg) or histamine (7 μg/kg), prior to a single intratracheal administration of the study drug (V0162), tiotropium (Spiriva®, Boehringer Ingelheim) or vehicle. Intratracheal administrations were performed using the Dry Powder Insufflator Model DP-4 (Penn-Century Inc). Successive i.v. injections of acetylcholine or histamine were performed 5, 10 and 15 min before the intratracheal administrations (to obtain basal values) and 5, 15, 30 and 60 min after the intratracheal administrations of either VO162or tiotropium. The experiment was not extended beyond 60 min since both the effects of V0162 and tiotropium reached a plateau, as previously shown for tiotropium [19].

### Ovalbumin-induced bronchoconstriction and lung inflammation in Guinea pigs

Ovalbumin-sensitized guinea pigs represent a relevant animal model to assess the effect of V0162 on both bronchoconstriction and inflammation [20]. Male Guinea pigs (250–300 g, 11–16 animals per group) were sensitized by bilateral i.p. injections of ovalbumin (100 μg) and aluminum hydroxide (100 mg) in 1 ml of physiological serum on Day 1, followed by a booster injection on Day 5 [20]. On Day 15, an ovalbumin challenge was carried out with a nebulized solution of ovalbumin (200 μg in 1 ml of physiological serum). Single intratracheal administrations of V0162 (50 μg), tiotropium (3 μg), budesonide (800 μg, Pulmicort®, Astrazeneca,) or vehicle were performed 15 min before the ovalbumin challenge, under a slight isoflurane anesthesia. Ventilatory parameters were measured before and one hour post-challenge, using whole body plethysmography (Emka, Technologies, Paris). The “enhanced pause” (PenH) AUC value, which represents the index of bronchopulmonary resistance, was calculated using the following formula (peak expiratory flow / peak inspiratory flow) x pause. Although we assume that the use of PenH to assess pulmonary function has been challenged, it is actually one of the classical parameter used in many publications [21]. The whole body plethysmography does not allow for residual volume measurement as performed in the Konzett and Rossler experiments on ventilated anesthetized animals.

Bronchoalveolar lavages (BAL) were performed after euthanasia 24 h post-challenge. A tracheotomy was performed and saline was gently infused into the lung; after recovery, samples were centrifuged and pellets suspended in 1 ml of saline. Total number of leukocytes was counted with ADVIA 2120i Hematology System (Siemens Diagnostics).

### Phase I/II study

This phase I/II combined study consisted of dose escalation study assessing the safety in healthy volunteers, followed by a proof-of-concept study assessing the efficacy and safety of V0162 in COPD patients (ClinicalTrials.gov identifier: NCT01348555).

### Subjects

Eligible subjects for the healthy volunteers study were males aged between 18 and 50 years, with a body mass index (BMI) ranging from 18 to 30 kg/m^2^.

Eligible patients for the COPD study were men or postmenopausal women aged between 40 and 75 years, BMI between 18 and 35 kg/m^2^, having a post-bronchodilator forced expiratory volume in 1 s (FEV_1_) ≥30 % and <80 % predicted, a post-bronchodilator FEV_1_/forced vital capacity (FVC) <70 %, with a smoking history of ≥ 10 pack-year and lung hyperinflation defined as a functional residual capacity (FRC) greater than 120 %.

Subjects with past or current history of asthma, or other significant respiratory conditions, or who had experienced an exacerbation within the last 6 weeks, were excluded from the study. Subjects with abnormal vital signs, or abnormal laboratory findings, or clinically relevant ECG abnormalities, or cardiovascular conditions prior to screening were also excluded from the study.

All subjects provided written informed consent prior to study participation. The study was conducted in France and Belgium. The protocol was approved in France by the “Comité de Protection des Personnes Ile de France VI Pitié Salpêtrière” (Approval n° 3–11) and the French National Agency for Medicine and Health Products Safety (Approval n° A110118-77), and in Belgium by the Institutional Review Board ZNA/OCMW of Antwerp (EC Approval n° 3945) and the Federal Agency for Medicines and Health Products (Approval n° 380756).

### Study design

**Fig. 1:**
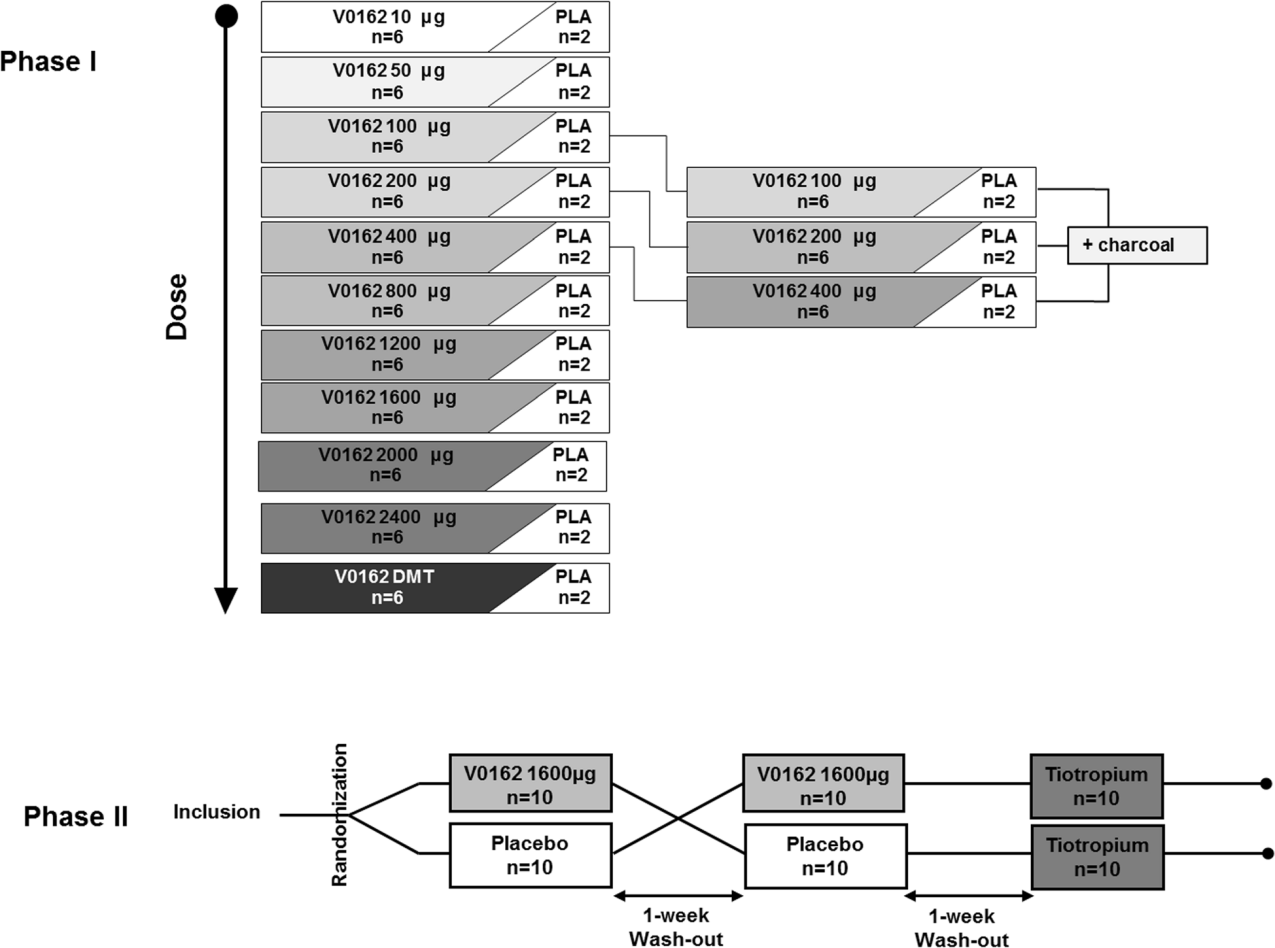
Phase I and Phase II study design

The healthy volunteers study was randomized, placebo-controlled, double blind with escalating-dose design. At each dose level, subjects were randomly assigned 3:1 to receive either a single-dose of V0162 (*n* = 6) or placebo (*n* = 2) (Fig. 1).

Additionally, study drug (V0162 or placebo) was co-administered with activated charcoal (Toxicarb®, SERB) to prevent the digestive absorption, as presented in Fig. 1.

The COPD study was randomized, placebo-controlled, double-blind, double-dummy cross-over study consisting of three periods. All patients were given both a single-dose of V0162 1600 μg and placebo in two alternating periods of time during the study (Fig. 1). The study drug was packed into hard capsules for use in dry powder inhalers (DPIs). In a third single-blind period, all patients received tiotropium (Spiriva® 18 μg delivered via HandiHaler®, Boehringer Ingelheim), the mainstay of COPD treatment. At this stage of development, it was decided to use a very easy-to-handle device, “the RS01 monodose” (Plastiape, Spa.). This DPI is a low resistance device reaching a pressure drop of 4 kPa at 100 L/min. Nevertheless, its performance when used to deliver V0162 could be optimized in the future, given that the fine particle dose (FPD) is around 15 % of the nominal dose. It might be compared with other marketed DPIs providing a FPD up to 45 % of the nominal dose [22]. Obviously, DPI performance should be improved in the next clinical development stages of V0162.

### Assessments

Pulmonary function tests (PFT) were performed at inclusion, over the first 12 h following treatment administration in healthy volunteers and over the first 32 h after treatment administration in COPD patients, and at the end-of-study visit. PFT consisted of spirometry (MasterScope®, Jaeger) in healthy volunteers and plethysmography (MasterScope Body®, Jaeger in France and BodyBox®, MediSoft in Belgium) in COPD patients, performed according to the American Thoracic Society/European Respiratory Society (ERS/ATS) guidelines. Along with PFT, dyspnea was evaluated by the patient using a 20 cm bidirectional visual analog scale (VAS) ranged from “-10 to +10”, with −10 for “Improvement in dyspnea”, 0 for “No dyspnea” and +10 “Intolerable dyspnea”. Blood sampling was performed to assess the pharmacokinetics (PK) of V0162 in healthy volunteers and in COPD patients.

In both studies, general safety parameters included AEs, vital signs, physical examination, clinical laboratory safety tests and electrocardiograms (12-lead ECG). In addition, 24-h holter (ECG) were performed in healthy volunteers.

### End points

In the healthy volunteers study, the primary objective was to assess the safety of V0162. Secondary end-points included PK parameters (C_max_, t_max,_ AUC_0-t_, AUC_0-∞_) and changes in FEV_1_.

In the COPD study, the primary objective was to evaluate the bronchodilator effect of V0162. The primary end-point was the change in the time-normalized area under the curve of FEV1 over the period from 30 min to 9 h post-dose (AUC30min/9 h) between V0162 and placebo. Time -normalized AUCs (expressed in mL) were calculated as AUC over a given period of time (expressed in mL*h) divided by the duration of the period (expressed in h). Other end-points were FEV1 AUC30min/9 h of tiotropium, trough FEV1, forced vital capacity (FVC), functional residual capacity (FRC), dyspnea and safety. Trough FEV_1_ was defined as the lowest value observed between 30 min and 32 h. Dyspnea was evaluated using the net incremental area under the curve (nIAUC), which represents the AUC below the baseline value.

### Statistical analysis and sample size calculation

In preclinical experiments evaluating induced-bronchoconstrition, a mixed model with treatment, as fixed factor and time as repeated measure, was performed. Time x treatment interactions were tested also. Baseline values were used as covariates (MIXED procedure in SAS R9.3). For the plethysmograph analyses, a one-way ANOVA followed by a Dunn’s test was performed.

The healthy volunteers study was an exploratory dose-escalation study; it was therefore conducted in a convenient population sample without prior sample size calculation.

For the COPD study, 20 patients were required to detect a mean difference of 150 mL in FEV_1_ AUC_30min/9h_ between V0162 and placebo (SD 200 mL; within-subject correlation coefficient 0.75) with 88 % of power at a two-sided significance level of 0.05.

Descriptive statistics, including mean ± standard error (SE) and changes from baseline were provided. In the healthy volunteers study, statistical analysis was performed on FEV1 change from baseline. In the COPD study, the FEV_1_ AUC_30min/9h_ was compared between V0162 and placebo in a mixed linear model for cross-over designs, as proposed by Kenward and Roger [23]. The adjusted means (LS mean) for treatment groups were provided with their 95 % CI. Given the study design, there were no comparisons planned between tiotropium and study drugs (V0162 or placebo).

## Results

### V0162 effects on acetylcholine- and histamine-induced bronchoconstriction in Guinea pigs

**Fig. 2:**
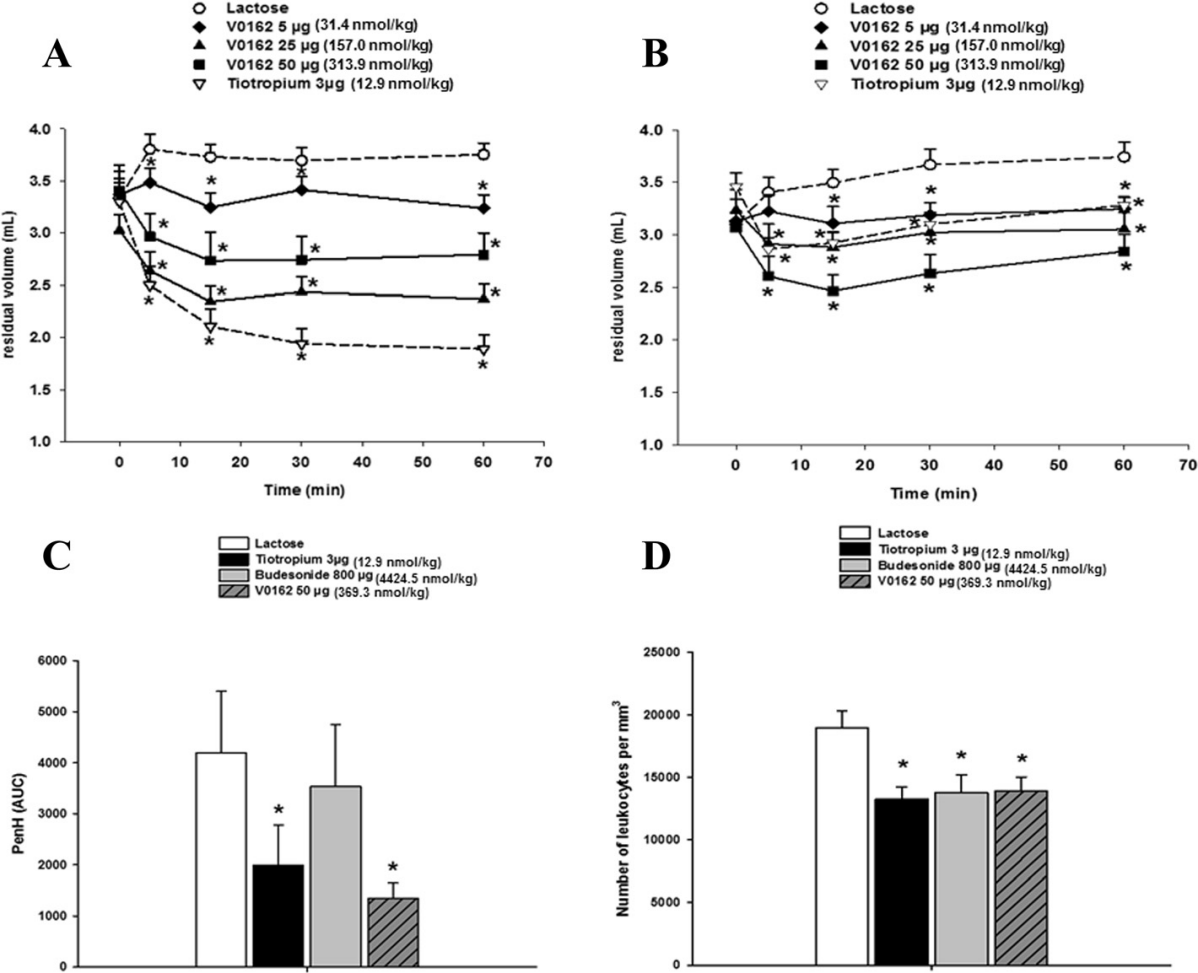
Bronchoprotection and anti-inflammatory effects of intratracheal V0162 single-dose in preclinical models. **a** Effect over time of V0162 and tiotropium on bronchoconstriction induced by acetylcholine in Guinea pigs, **b** Effect over time of V0162 and tiotropium on bronchoconstriction induced by histamine in Guinea pigs. Data are expressed as mean ± SEM. *p < 0.05 vs. vehicle (A and B, mixed model with treatment, as fixed factor and time as repeated measure. Baseline values were used as covariates). **c** Effect of V0162, tiotropium and budesonide on pulmonary resistance in ovalbumin-sensitized Guinea pigs. **d** Effect of V0162, tiotropium and budesonide on the total number of leukocytes in BAL performed 24 h post-challenge. Data are expressed as mean ± SEM. *p < 0.05 vs. vehicle (**c** and **d**, one-way ANOVA followed by a Dunn’s test)

A Guinea pig model of bronchoconstriction induced by acetylcholine or histamine was used to assess the anti-bronchoconstrictor effect of V0162. In untreated Guinea pigs, residual volume (RV) was generally between 1.4 and 2.0 ml (data not shown). During acetylcholine- and histamine-induced bronchoconstriction, the increase in the RV (up to 3.8–4 mL) was prevented and even a dose-dependent decrease of RV was obtained after the single intratracheal administration of V0162 (Fig. 2a, b). The maximal effect was reached at 15 min with 25 μg of V0162 after acetylcholine-induced bronchoconstriction (a 53 % significant decrease from baseline, *p* < 0.05), and with 50 μg of V0162 in histamine-induced bronchoconstriction (46 % significant decrease from baseline, *p* < 0.05). The bronchodilator effect of V0612 was maintained up to 60 min. Tiotropium (3 μg) exhibited anti-bronchoconstrictor properties, slightly larger than V0162 facing the acetylcholine challenge, but of lower magnitude than V0162 during the histamine challenge (Fig. 2). At 60 min, the mean inhibition of the acetylcholine-induced bronchoconstriction was 14 %, 37 % and 26 % for V0162 5 μg, 25 μg and 50 μg respectively, and 50 % for tiotropioum 3 μg, compared with vehicle (lactose). The mean inhibition of the histamine-induced bronchoconstriction was 13 %, 18 % and 24 % by V0162 5 μg, 25 μg and 50 μg, respectively, and 12 % by tiotropium 3 μg, compared with vehicle.

### V0162 effects in ovalbumin-induced bronchoconstriction in Guinea pig

A single intratracheal administration of V0162 in sensitized animals decreases significantly the ovalbumin-induced bronchoconstriction compared with vehicle (Fig. 2-c). The PenH AUC was reduced by 68 %, almost half an hour after a single intratracheal administration of V0162 at 50 μg, and a comparable effect was obtained with tiotropium (a decrease of 53 % in PenH AUC). Budesonide did not reduce significantly the intensity and the duration of the bronchoconstriction.

Twenty four hours post-challenge, vehicle-treated Guinea pigs showed a massive leukocyte infiltration (18 985 ± 1 368/ mm^3^ in BAL fluid). This inflammatory response was partially prevented by V0162 (50 μg), tiotropium (3 μg) and budesonide (800 μg) in a similar manner (Fig. 2-d). A reduction of 27 %, 30 % and 27 % was observed in total leukocytes with V0162, tiotropium and budesonide, respectively.

### Enrollment and patient disposition

In the healthy volunteers study, 153 male subjects were screened and 88 were randomized (Fig. 1). No significant differences were found in baseline characteristics between dose cohorts In the COPD study, 37 patients were screened and 20 were randomized (Fig. 1). Subjects’ characteristics are presented in Table 1. There was no premature withdrawal throughout all the study.

**Table 1:** Demographics and baseline characteristics

	Healthy volunteers *n* = 88	COPD patients *n* = 20
Male	88	12
Age, years	31.3 ± 8.8	60.4 ± 5.5
BMI, kg/m^2^	24.4 ± 2.9	24.5 ± 4.4
Time since diagnosis, years	NA	5.9 ± 4.4 (0.1–15.8)
Never smokers	75	-
Former smokers	13	20
Tobacco consumption, pack-years	NA	47.5 ± 25.1
FEV1, L	4.15 ± 0.53	1.23 ± 0.53
FEV1 (% predicted)	97.71 ± 10.28	43.40 ± 15.16
FVC, L	5.05 ± 0.66	2.76 ± 0.83
FVC (% predicted)	118.95 ± 13.76	98.03 ± 20.66
FEV1/FVC, %	82.43 ± 5.52	43.84 ± 10.41
FRC (% predicted)	-	180.28 ± 44.10
RV (% predicted)	-	220.71 ± 62.15
RV/TLC (% predicted)	-	164.86 ± 22.15

Results are presented by mean ± SD (min-max) or number of patients (n)

*NA* not applicable

### Safety

In both studies, there were no serious AEs reported.

In the healthy volunteers study, the maximal tolerated dose (MTD) was not reached. Taking into account the large number of inhalations, the dose escalation was stopped at 2400 g (12 capsules/24 inhalations) which was considered as the MAD with the current study drug formulation (200 μg per capsule). Thirteen AEs were reported in 9 healthy volunteers (Table 2). Of these, 9 events were considered as related to the study drug in 7 subjects.

**Table 2:** Treatment-emergent AEs in healthy volunteers and COPD patients

	COPD patients						Healthy volunteers			
	Placebo (*n* = 20)		V0162 (*n* = 20)		Tiotropium (*n* = 20)		Placebo (*n* = 22)		V0162 (*n* = 66)	
Adverse event	Event n	Patients n (%)	Event n	Patients n (%)	Event n	Patients n (%)	Event n	Patients n (%)	Event n	Patients n (%)
Total	28	8 (40)	42	16 (80)	23	12 (60)	6	4 (18)	7	5 (8)
Cough	7	4 (20)	14	10 (50)	7	6 (30)	1	1 (4.5)	3	3^a^ (4.5)
Dyspnea	5	4 (20)	8	7 (35)	6	4 (20)	-	-	-	-
Productive Cough	7	4 (20)	6	6 (30)	4	4 (20)	-	-	-	-
Headache	3	3 (15)	3	3 (15)	3	3 (15)	3^b^	3 (13.6)	-	-
Throat Irritation	1	1 (5)	3	3 (15)	1	1 (5)	1	1 (4.5)	-	-
Wheezing	1	1 (5)	2	1 (5)	1	1 (5)	-	-	-	-
Pruritus	-	-	3	3 (15)	-	-	-	-	1	1^c^ (1.5)
Abdominal pain	1	1 (5)	-	-	-	-	-	-	1	1^c^ (1.5)
Angina Pectoris	-	-	-	-	1	1 (5)	-	-	-	-
Dizziness	1	1 (5)	-	-	-	-	-	-	-	-
Erysipelas	1	1 (5)	-	-	-	-	-	-	-	-
Erythema	1	1 (5)	-	-	-	-	-	-	-	-
Fatigue	-	-	1	1 (5)	-	-	-	-	-	-
Diarrhea	-	-	1	1 (5)	-	-	-	-	-	-
Dry Mouth	-	-	1	1 (5)	-	-	-	-	-	-
Nausea	-	-	-	-	-	-	1	1 (4.5)	1	1^d^ (1.5)
Epistaxis	-	-	-	-	-	-	-	-	1	1^e^ (1.5)

A patient with multiple AEs is counted only once

^a^2 subjects at 1200 μg and 1 at 2400 μg

^b^1 subject had received placebo + charcoal

^c^at 1200 μg

^d^at 2000 μg

^e^at 800 μg

In the COPD study, 93 AEs were reported in 17 patients: 42 experienced with V0162, 28 with placebo and 23 with tiotropium (Table 2). Twenty-six AEs were related to the treatment in 9 COPD patients.

Most frequent AEs reported with V0162 were cough (10/20 in patients with COPD and 3/66 in health volunteers), dyspnea (7/20), productive cough (6/20) and pruritus (3/20 and 1/66). All these experienced AEs were mild to moderate in severity; none had led to study drug discontinuation.

In both healthy volunteers and COPD patients, no relevant changes from baseline were observed in physical examination, vital signs, body weight, 12-lead ECG parameters or in laboratory parameters. Although some COPD patients presented abnormalities on QTcF regardless of the study product, the only one clinically significant abnormality was already present at baseline, and consequently was not related to the study drug. V0162 did not led to cardiac abnormalities in healthy volunteers, nor in COPD patients. As shown below in PK results, the C_max_ was very low (2 ng/mL), and the compound was quickly eliminated regardless of the dose, thus preventing systemic anticholinergic side effects.

### Pharmacodynamics

In healthy volunteers study, there was no significant change in FEV1 between V0162 and placebo. However, an increase in FEV_1_ was observed with V0162 1200 μg compared with placebo (0.84 L, 95 % CI [−15; 183]); the difference not reaching the statistical significance (*p* = 0.094).

**Fig. 3:**
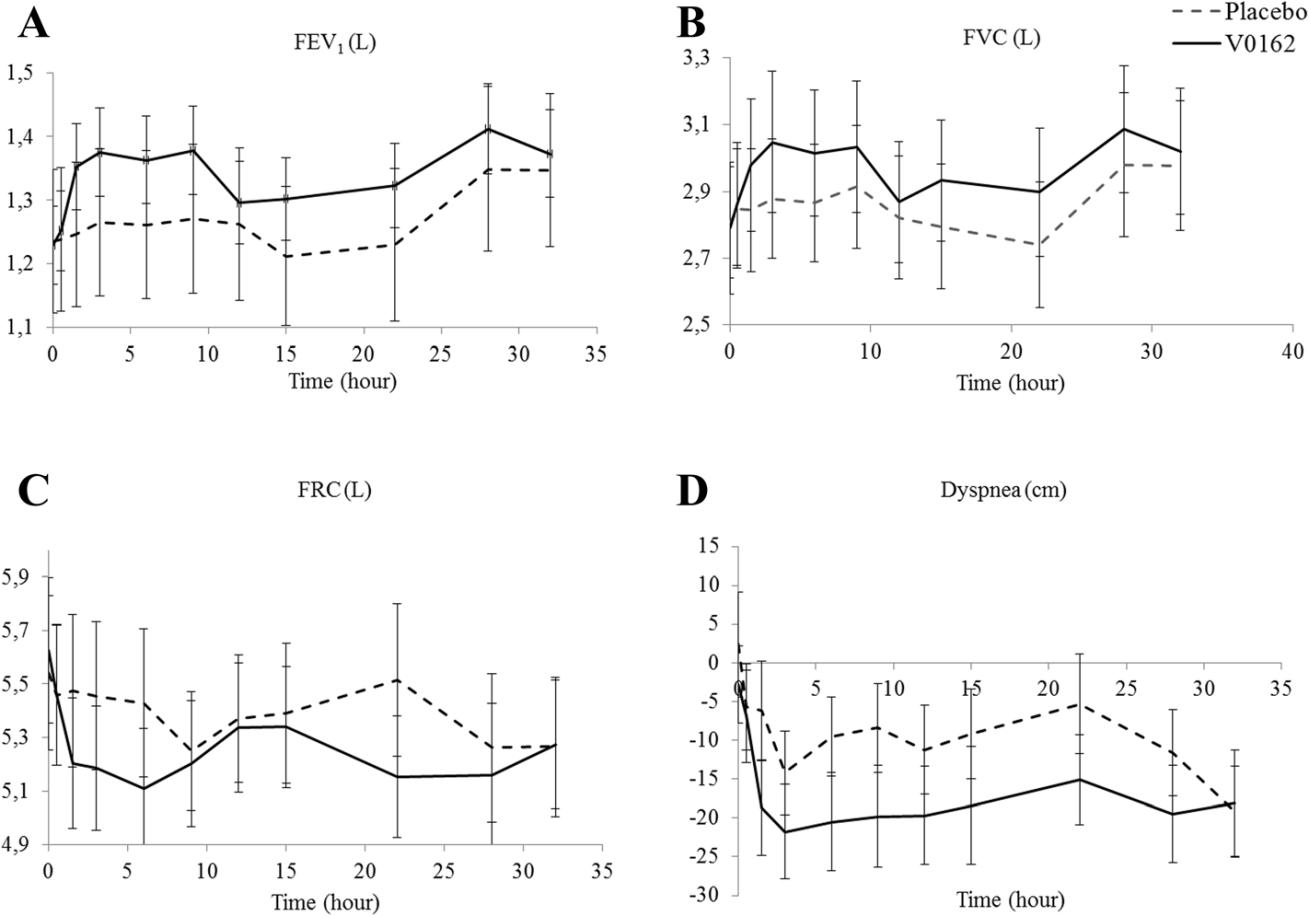
**a** Mean values and standard error (SE) of FEV_1,_ **b** mean values and SE of FVC, **c** mean values and SE of FRC, **d** change from baseline of dyspnea (VAS mm) over time (from 0 to 32 h) and SE, in placebo- and V0162-treated COPD patients. (V0162 1600 μg, *n* = 20)

In the COPD study, V0162 improved significantly the FEV1 AUC30min/9 h of 0.10 ± 0.03 L (95%CI: 0.04–0.16), compared with placebo. This was also the peak effect observed at 9 h. V0162 exhibited a long-lasting bronchodilator effect maintained up to 32 h after inhalation (Fig. 3-a). FEV_1_ AUC_30min/22h,_ FEV_1_ AUC_30min/28h_ and FEV_1_ AUC_30min/32h_ changes from placebo were of 0.09 ± 0.03 L, 0.08 ± 0.03 L and 0.09 ± 0.03 L, respectively (Table 3). The difference in trough FEV1 between V0162 and placebo was 0.08 ± 0.03 L, *p* = 0.016.

**Table 3:** Pharmacodynamic parameters in COPD patients treated with V0162 versus placebo

COPD patients			
	V0162 (*n* = 20)	Placebo (*n* = 20)	*p*-value
FEV1			
AUC_30min/6h,_ L	1.36 ± 0.13	1.26 ± 0.11	0.004
AUC_30min/9h,_ L	1.36 ± 0.12	1.26 ± 0.12	0.003
AUC_30min/22h,_ L	1.33 ± 0.12	1.24 ± 0.12	0.006
AUC_30min/28h,_ L	1.34 ± 0.12	1.25 ± 0.12	0.007
AUC_30min/32h,_ L	1.35 ± 0.12	1.27 ± 0.12	0.006
Trough FEV1, L	1.19 ± 0.11	1.11 ± 0.11	0.016
FVC			
AUC_30min/6h,_ L	3.01 ± 0.20	2.86 ± 0.18	0.019
AUC_30min/9h,_ L	3.01 ± 0.19	2.87 ± 0.19	0.011
AUC_30min/22h,_ L	2.96 ± 0.18	2.83 ± 0.18	0.017
AUC_30min/28h,_ L	2.96 ± 0.19	2.84 ± 0.18	0.013
AUC_30min/32h,_ L	2.98 ± 0.19	2.85 ± 0.19	0.014
FRC			
AUC_30min/6h,_ L	5.24 ± 0.23	5.45 ± 0.29	<0.001
AUC_30min/9h,_ L	5.25 ± 0.25	5.51 ± 0.25	<0.001
AUC_30min/22h,_ L	5.34 ± 0.24	5.52 ± 0.24	0.006
AUC_30min/28h,_ L	5.21 ± 0.24	5.43 ± 0.29	0.003
AUC_30min/32h,_ L	5.22 ± 0.24	5.41 ± 0.29	0.003
Dyspnea			
nIAUC_30min/6h,_ mm	- 17.79 ± 5.26	- 11.44 ± 5.27	0.161
nIAUC_30min/9h,_ mm	- 18.08 ± 5.31	- 10.93 ± 5.31	0.084
nIAUC_30min/22h,_ mm	- 17.45 ± 5.97	- 9.72 ± 5.97	0.054
nIAUC_30min/28h,_ mm	- 17.33 ± 6.04	- 9.43 ± 6.04	0.057
nIAUC_30min/32h,_ mm	- 17.45 ± 6.03	- 10.193 ± 6.03	0.078

Results are presented by mean ± SE

A similar pattern was observed for FVC and FRC. The mean values for FVC and FRC over time are presented in Fig. 3b, c, respectively. Compared with placebo, V0162 had statistically significant improved FVC AUC30min/9 h, FVC AUC30min/22 h and FVC AUC30min/32 h (Table 3). The V0162 effect on FRC AUC30min/9 h and AUC30min/22 h showed a significant decrease of 0.262 L (*p* < 0.001) and 0.177 L (*p* = 0.006) compared with placebo, respectively (Table 3). No significant reduction in VR was observed with V0162.

V0162 tended to reduce dyspnea ratings as compared with placebo, without reaching statistical significance: the decrease in nIAUC30min/9 h and nIAUC30min/22 h was of −7.15 ± 3.9 mm (*p* = 0.084) and −7.7 ± 3.75 mm (*p* = 0.054, respectively (Table 3 & Fig. 3-e)).

Tiotropium showed a pronounced bronchodilator effect in patients with COPD: LS mean of FEV1 AUC30min/9 h was 1.50 ± 0.12 L. At trough, tiotropium FEV1 was 1.32 L. This benefit was maintained up to 32 h: FEV1 AUC30min/22 h, FEV1 AUC30min/28 h and FEV1 AUC30min/32 h were 1.47 L, 1.46 L and 1.7 L, respectively. FVC AUC30min/9 h and AUC30min/22 h were 3.28 L and 3.21 L, respectively. Like V0162, tiotropium tended to reduce dyspnea as compared with placebo, without reaching a statistical significance: nIAUC30min/9 h and nIAUC30min/22 h were −16.2 ± 5.2 mm (NS) and −16.2 ± 5.8 mm (NS), respectively.

### Pharmacokinetics

**Fig. 4:**
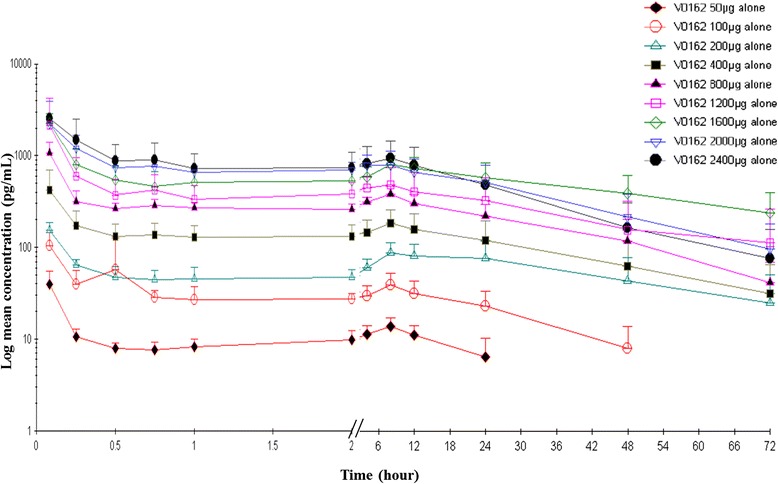
Mean plasma concentration over 72 h after a single dose of V0162 50 μg, 100 μg, 200 μg, 400 μg, 800 μg, 1200 μg, 1600 μg, 2000 μg, 2400 μg in healthy volunteers s (log/linear scale)

PK parameters are summarized in Table 4. Following a single-dose of V0162 administered by oral inhalation in healthy volunteers, the maximum plasma level (C_max_) was reached between 0.08 and 0.10 h post-dose, followed by a rapid decrease (Fig. 4). Second increase in plasma levels was observed between 0.75 h and 8 h post-dose. This later increase was not observed when digestive absorption was blocked with activated charcoal. A long elimination phase was observed, with mean t_1/2_ varying between 17.4 h and 36.9 h post-dose. The AUCs ratio of V0162 + charcoal/V0162 alone suggested that digestive absorption contribute to 42–48 % of the overall V0162 absorption, beside the pulmonary absorption. C_max_ and AUC_0-∞_ were nonlinear across the range of tested doses. In cohorts ranging from 50 to 2400 μg, when the dose increased by two-fold, C_max_ and AUC_0-∞_ increased by 2.17 and 2.03-fold, respectively. There was no significant statistical difference in PK parameters between V0162 2400 μg alone and combined with ipratropium/fenoterol.

**Table 4:** V0162 pharmacokinetic parameters for different dose cohorts in healthy volunteers and in COPD patients

V0162 dose	t_max_ (h)	C_max_ (pg/mL)		AUC_0-t_ (h.pg/mL)		t_1/2_ (h)	
Healthy volunteers	Median	Mean	SD	Mean	SD	Mean	SD
10 μg	nc	nc	nc	nc	nc	nc	nc
50 μg	0.08	40	14	555	790	26	13
100 μg	0.08	108	41	1872	1981	19	0.69
200 μg	0.08	149	33	4578	2633	28	11
400 μg	0.08	431	268	6529	3774	23	8.08
800 μg	0.08	1030	311	17670	13735	19	4.88
1200 μg	0.08	2180	2030	17646	12297	20	11
1600 μg	0.08	2270	618	32692	13447	36	15
2000 μg	0.08	2970	1800	41192	37151	19	3.55
2400 μg	0.10	2840	1040	26820	16211	17	3.40
100 μg + charcoal	0.08	98	65	560	250	21	3.99
200 μg + charcoal	0.08	81	28.8	1483	411	32	13
400 μg + charcoal	0.08	359	216	3541	1871	27	8.32
2400 μg + ipratropium/fenoterol	0.11	2600	824	31200	9941	19	4.17
COPD patients							
1600 μg	0.08	1040	577	13872.8	6461	nc	nc

*nc* not calculated (more than 50 % of plasma values were below the lower limit of quantification (5.00 pg/mL)

Like in healthy volunteers, C_max_ in COPD patients was reached at 0.08 h post-dose and a second peak was observed, followed by a long elimination phase. Mean C_max_ was 54 % lower in COPD patients compared with healthy volunteers.

## Discussion

Our preclinical studies showed that V0162 not only reverses bronchoconstriction induced either by acetylcholine or histamine, but also prevents ovalbumin-induced bronchoconstriction and lung inflammation. In animal models, particularly in ovalbumin-sensitized animals, specific muscarinic antagonists have been shown to exert anti-inflammatory activities with reduction in airway smooth muscle thickening and fibrosis, airway hyper-responsiveness, mucous gland hypertrophy, Th2 cytokine production and eosinophil infiltration [24–27]. In a recent large clinical study, tiotropium used as add-on therapy reduced the risk of severe exacerbation in asthmatic patient who have symptoms despite standard treatments [28]. Based on these results, tiotropium has been recently approved by the regulatory authorities in the EU, as an add-on maintenance bronchodilator treatment in adult asthmatic patients currently treated with the maintenance combination of inhaled corticosteroids and long-acting beta2-agonists, and who experienced one or more severe exacerbations in the previous year.

The efficacy of V0162 in reversing histamine-induced bronchoconstriction and its anti-inflammatory effect in the ovalbumin-sensitized animal model are also likely related to the antihistaminic properties of mequitazine, the parent compound. Antihistamines have been actually shown to exert anti-inflammatory activities in allergen-sensitized animal models [29–33]. A meta-analysis of clinical trials in asthma has suggested that antihistamines induce bronchodilation in comparison with placebo; but this effect was of limited magnitude and the systemic effects observed after repeated use of antihistamines do not support the use of these medications alone in the treatment of asthma [34]. Through an inhalation route, the risk of systemic adverse events is very low with a compound like V0162 as shown in the present PK and safety results.

The blockade of H1 histamine receptors by V0162 in the airways is likely to induce a weak bronchodilating effect, in addition to the bronchodilating effect related to the blockade of M3 muscarinic receptors. The potential additive (even synergistic) bronchodilating effect of compounds acting by different mechanisms on the airway smooth tone has been documented previously [35–38]. Altogether, the anti-histaminic properties of V0162 could confer benefit particularly in asthma, in addition to the main anticholinergic-related bronchodilation observed in COPD patients and to the potential anticholinergic-related anti-inflammatory activities.

The properties of V0162 were similar to those observed with tiotropium but at higher tested doses. At similar doses, V0162 had less prominent effect than tiotropium on bronchoconstriction induced by acetylcholine. This difference could be explained by the fact that the affinity of V0162 at muscarinic receptors according to the affinity of mequitazine [16, 39] may be about 100-fold lower than that of tiotropium.

Our clinical results showed that a single-dose of V0162 induces sustained bronchodilation in patients with COPD, as evidenced by statistically significant improvement in FEV_1_ compared with placebo, maintained up to 32 h [40–42]. This improvement was clinically relevant since the minimal clinically important difference of 100 ml was achieved at the peak effect. [40–42]. The results observed were slightly different from the assumed hypothesis; the observed difference in FEV1 AUC30_min/9 h_ was actually smaller and the variability was lower. However, these findings led to clinically and statistically significant results. The positive impact on the FEV_1_ was consistent with the benefit observed on the reduction of hyperinflation and on dyspnea as assessed by patients. The observed effect of tiotropium was in accordance with those found in literature [8]. This finding confirms the reliability of the observed effects. No direct comparisons were performed between tiotropium and V0162 due to the study design.

The tested dose throughout the COPD study was 1600 μg, although the MAD was 2400 μg in the dose-escalation study. Lower dose was considered to minimize the treatment burden, given the relatively high number of capsules (200 μg each) and inhalations (2 per capsule). The purpose was to assess the bronchodilator properties of the compound. However, the performance of the aerodynamic properties remains under investigation. Aerosol particle size emitted from the DPI is one of the most important variables in defining drug delivery [43, 44]. Fine particles with aerodynamic diameter ≤ 5 μm are essential to ensure optimal drug deposition and distribution. Currently, the fine particle dose (FPD), defined as the dose of the fine particles in the emitted aerosol, is quiet low (almost 15 % of the nominal dose with the current DPI). The use of an optimized DPI can increase the FDP up to 45 % [45] (+diffuse study), leading to an increase in pulmonary deposition and potentially to an improvement in the deposition in distal airways according to the particle sizes [46–48].

The administration of activated charcoal combined with V0162 showed that digestive absorption contributes significantly to the overall bioavailability: V0162 is absorbed at the gastrointestinal tract, with approximately 42 to 48 % of the overall absorption. A rapid pulmonary absorption was observed few minutes after inhalation (which corresponded also to the C_max_) and was followed by a digestive absorption observed 8 to 12 h later.

Although maximal plasmatic concentration was reached rapidly, V0162 exhibited low systemic exposure (at the picomolar range) with a long-lasting half-life (up to 36 h). Topical administration *via* the inhalation route allows a rapid and potent local activity in the bronchial tree, without leading to high systemic exposure. Consequently potential systemic adverse events, including cholinergic side effects and cardiovascular events, may be prevented. Mean maximal plasmatic concentration was almost two-fold lower in COPD patients compared with healthy volunteers. This finding may be due to the underlying disease. In fact, airway narrowing and reduction in the inspiratory flow may alter lung deposition [43].

As a result, V0162 was well tolerated with a good overall safety profile. No serious AEs were reported and all AEs were mild in severity. Most frequent AEs included productive and non-productive cough, dyspnea and pruritus. These observed AEs are consistent with previous reports on inhaled bronchodilators; common AEs are generally a result of the patient’s preexisting condition and of the route of administration [49–51]. There was no dose–response relationship between V0162 and AEs. Laboratory findings, vital signs and ECG observations were unchanged during the study. No particular electrocardiographic change was observed. The V0162 plasma levels appear 50-fold lower than the concentration threshold (330 nM) that may cause hERG cardiac toxicity.

Given the exploratory nature of this study, this latter is potentially limited by its sample size, the single-dose setting, the short-duration, and the use of a non-optimized DPI. Nevertheless, it is the first clinical investigation which asses the safety and efficacy of V0162 in human. Larger phase II studies with longer study duration, other study designs and optimized formulations are on-going.

## Conclusions

Inhaled V0162, given at 1600 μg single-dose, resulted in statistically significant long-lasting bronchodilation that reached the minimal clinically important difference threshold for FEV1 at the peak effect, and positive safety profile in patients with moderate-to-severe COPD. At this stage of development, the choice of the tested dose was based on safety data, and does not correspond to the minimal effective dose. A phase II dose-finding study is required to estimate the minimal effective dose. Given the activity of V0162 as an antagonist of muscarinic receptors and its additional activities (e.g. anti-histamine activity), this compound would be of interest for the treatment of COPD and potentially also of asthma as recently shown with tiotropium, a pure LAMA. More clinical investigations in these two respiratory diseases are yet needed.

## Abbreviations

AUC: Area under the curve
AEs: Adverse events
BAL: Bronchoalveolar lavages
BMI: Body mass index
CI: Confidence interval
Cmax: Maximum plasma level
COPD: Chronic obstructive pulmonary disease
DPI: Dry powder inhaler
ECG: Electrocardiogram
FEV_1_: Forced expiratory volume
FVC: Functional vital capacity
FRC: Functional residual capacity
GOLD: Global initiative for chronic obstructive lung disease
i.v: Intravenous
LAMA: Long-acting muscarinic receptor antagonist
MAD: Maximum administrated dose
MTD: Maximum tolerated dose
nIAUC: Net incremental area under the curve
NS: Not significant
PenH: Enhanced pause
PFT: Pulmonary function tests
PK: Pharmacokinetics
RV: Residual volume
t_max_: Time to reach maximum plasma level
VAS: Visual analog scale

## References

[1] Lopez AD, Shibuya K, Rao C, Mathers CD, Hansell AL, Held LS, Schmid V, Buist S (2006). Chronic obstructive pulmonary disease: current burden and future projections. Eur Respir J.

[2] Global strategy for the diagnosis, management, and prevention of COPD, Global Initiative for Chronic Obstructive Lung Disease (GOLD) 2015. Available from: http://www.goldcopd.org/10.1164/ajrccm.163.5.2101039 11316667

[3] Koumis T, Samuel S (2005). Tiotropium bromide: a new long-acting bronchodilator for the treatment of chronic obstructive pulmonary disease. Clin Ther.

[4] Casaburi R, Briggs DD, Donohue JF, Serby CW, Menjoge SS, Witek TJ (2000). The spirometric efficacy of once-daily dosing with tiotropium in stable COPD: a 13-week multicenter trial. The US Tiotropium Study Group. Chest.

[5] Casaburi R, Mahler DA, Jones PW, Wanner A, San PG, ZuWallack RL, Menjoge SS, Serby CW, Witek T (2002). A long-term evaluation of once-daily inhaled tiotropium in chronic obstructive pulmonary disease. Eur Respir J.

[6] Littner MR, Ilowite JS, Tashkin DP, Friedman M, Serby CW, Menjoge SS, Witek TJ (2000). Long-acting bronchodilation with once-daily dosing of tiotropium (Spiriva) in stable chronic obstructive pulmonary disease. Am J Respir Crit Care Med.

[7] O’Donnell DE, Fluge T, Gerken F, Hamilton A, Webb K, Aguilaniu B, Make B, Magnussen H (2004). Effects of tiotropium on lung hyperinflation, dyspnoea and exercise tolerance in COPD. Eur Respir J.

[8] Decramer M, Celli B, Kesten S, Lystig T, Mehra S, Tashkin DP (2009). Effect of tiotropium on outcomes in patients with moderate chronic obstructive pulmonary disease (UPLIFT): a prespecified subgroup analysis of a randomised controlled trial. Lancet.

[9] Tashkin DP, Celli B, Senn S, Burkhart D, Kesten S, Menjoge S, Decramer M (2008). A 4-year trial of tiotropium in chronic obstructive pulmonary disease. N Engl J Med.

[10] Celli B, Decramer M, Kesten S, Liu D, Mehra S, Tashkin DP (2009). Mortality in the 4-year trial of tiotropium (UPLIFT) in patients with chronic obstructive pulmonary disease. Am J Respir Crit Care Med.

[11] Cheyne L, Irvin-Sellers MJ, White J (2013). Tiotropium versus ipratropium bromide for chronic obstructive pulmonary disease. Cochrane Database Syst Rev.

[12] Cazzola M, Page C, Matera MG (2013). Long-acting muscarinic receptor antagonists for the treatment of respiratory disease. Pulm Pharmacol Ther.

[13] Cope S, Donohue JF, Jansen JP, Kraemer M, Capkun-Niggli G, Baldwin M, Buckley F, Ellis A, Jones P (2013). Comparative efficacy of long-acting bronchodilators for COPD - a network meta-analysis. Respir Res.

[14] Karabis A, Lindner L, Mocarski M, Huisman E, Greening A (2013). Comparative efficacy of aclidinium versus glycopyrronium and tiotropium, as maintenance treatment of moderate to severe COPD patients: a systematic review and network meta-analysis. Int J Chron Obstruct Pulmon Dis.

[15] Kew KM, Dias S, Cates CJ (2014). Long-acting inhaled therapy (beta-agonists, anticholinergics and steroids) for COPD: a network meta-analysis. Cochrane Database Syst Rev.

[16] Stanton T, Bolden-Watson C, Cusack B, Richelson E (1993). Antagonism of the five cloned human muscarinic cholinergic receptors expressed in CHO-K1 cells by antidepressants and antihistaminics. Biochem Pharmacol.

[17] Kubo N, Shirakawa O, Kuno T, Tanaka C (1987). Antimuscarinic effects of antihistamines: quantitative evaluation by receptor-binding assay. Jpn J Pharmacol.

[18] Konzett H, Rössler R (1940). Versuchsanordnung zu Untersuchungen an der Bronchiàlmuskulatur. Arch Exp Pathol Pharmakol.

[19] Villetti G, Bergamaschi M, Bassani F, Bolzoni PT, Harrison S, Gigli PM, Janni A, Geppetti P, Civelli M, Patacchini R (2006). Pharmacological assessment of the duration of action of glycopyrrolate vs tiotropium and ipratropium in guinea-pig and human airways. Br J Pharmacol.

[20] Wu ZX, Zhou D, Chen G, Lee LY (2000). Airway hyperresponsiveness to cigarette smoke in ovalbumin-sensitized guinea pigs. Am J Respir Crit Care Med.

[21] Halloy D, Cambrier C, Kirschvink N, Gustin P (2004). Experimental assessment of pulmonary function in pigs. Ann Med Vet.

[22] Johal B, Howald M, Fischer M, Marshall J, Venthoye G (2013). Fine particle profile of fluticasone propionate/formoterol fumarate versus other combination products: the DIFFUSE study. Comb Prod Ther.

[23] Kenward MG, Roger JH (1997). Small sample inference for fixed effects from restricted maximum likelihood. Biometrics.

[24] Buels KS, Jacoby DB, Fryer AD (2012). Non-bronchodilating mechanisms of tiotropium prevent airway hyperreactivity in a guinea-pig model of allergic asthma. Br J Pharmacol.

[25] Cui Y, Devillier P, Kuang X, Wang H, Zhu L, Xu Z, Xia Z, Zemoura L, Advenier C, Chen H (2010). Tiotropium reduction of lung inflammation in a model of chronic gastro-oesophageal reflux. Eur Respir J.

[26] Ohta S, Oda N, Yokoe T, Tanaka A, Yamamoto Y, Watanabe Y, Minoguchi K, Ohnishi T, Hirose T, Nagase H (2010). Effect of tiotropium bromide on airway inflammation and remodelling in a mouse model of asthma. Clin Exp Allergy.

[27] Xu ZP, Devillier P, Xu GN, Qi H, Zhu L, Zhou W, Hou LN, Tang YB, Yang K, Yu ZH (2013). TNF-alpha-induced CXCL8 production by A549 cells: involvement of the non-neuronal cholinergic system. Pharmacol Res.

[28] Kerstjens HA, Engel M, Dahl R, Paggiaro P, Beck E, Vandewalker M, Sigmund R, Seibold W, Moroni-Zentgraf P, Bateman ED (2012). Tiotropium in asthma poorly controlled with standard combination therapy. N Engl J Med.

[29] Beermann S, Glage S, Jonigk D, Seifert R, Neumann D (2012). Opposite effects of mepyramine on JNJ 7777120-induced amelioration of experimentally induced asthma in mice in sensitization and provocation. PLoS One.

[30] Blumchen K, Gerhold K, Thorade I, Seib C, Wahn U, Hamelmann E (2004). Oral administration of desloratadine prior to sensitization prevents allergen-induced airway inflammation and hyper-reactivity in mice. Clin Exp Allergy.

[31] Bryce PJ, Geha R, Oettgen HC (2003). Desloratadine inhibits allergen-induced airway inflammation and bronchial hyperresponsiveness and alters T-cell responses in murine models of asthma. J Allergy Clin Immunol.

[32] Llupia J, Gras J, Llenas J (2003). Comparative antiallergic effects of second-generation H1-antihistamines ebastine, cetirizine and loratadine in preclinical models. Arzneimittelforschung.

[33] Tuncel T, Karaman M, Firinci F, Uysal P, Kiray M, Bagriyanik AH, Yilmaz O, Karaman O, Uzuner N (2013). The effect of rupatadine on lung histopathology in a murine model of chronic asthma. J Asthma.

[34] Van Ganse E, Kaufman L, Derde MP, Yernault JC, Delaunois L, Vincken W (1997). Effects of antihistamines in adult asthma: a meta-analysis of clinical trials. Eur Respir J.

[35] Cazzola M, Calzetta L, Page CP, Rogliani P, Facciolo F, Gavalda A, Matera MG (2014). Pharmacological characterization of the interaction between aclidinium bromide and formoterol fumarate on human isolated bronchi. Eur J Pharmacol.

[36] Calzetta L, Page CP, Spina D, Cazzola M, Rogliani P, Facciolo F, Matera MG (2013). Effect of the mixed phosphodiesterase 3/4 inhibitor RPL554 on human isolated bronchial smooth muscle tone. J Pharmacol Exp Ther.

[37] Matera MG, Calzetta L, Parascandolo V, Curradi G, Rogliani P, Cazzola M (2009). Relaxant effect of brain natriuretic peptide in nonsensitized and passively sensitized isolated human bronchi. Pulmon Pharmacol Ther.

[38] Matera MG, Calzetta L, Passeri D, Facciolo F, Rendina EA, Page C, Cazzola M, Orlandi A (2011). Epithelium integrity is crucial for the relaxant activity of brain natriuretic peptide in human isolated bronchi. Br J Pharmacol.

[39] Martinez-Mir I, Estan L, Rubio E, Morales-Olivas FJ (1988). Antihistaminic and anticholinergic activities of mequitazine in comparison with clemizole. J Pharm Pharmacol.

[40] Donohue JF (2005). Minimal clinically important differences in COPD lung function. COPD.

[41] Meyer RJ (2005). U.S. regulatory perspective on the minimal clinically important difference in chronic obstructive pulmonary disease. COPD.

[42] Cazzola M, MacNee W, Martinez FJ, Rabe KF, Franciosi LG, Barnes PJ, Brusasco V, Burge PS, Calverley PM, Celli BR (2008). Outcomes for COPD pharmacological trials: from lung function to biomarkers. Eur Respir J.

[43] Labiris NR, Dolovich MB (2003). Pulmonary drug delivery. Part I: physiological factors affecting therapeutic effectiveness of aerosolized medications. Br J Clin Pharmacol.

[44] Labiris NR, Dolovich MB (2003). Pulmonary drug delivery. Part II: the role of inhalant delivery devices and drug formulations in therapeutic effectiveness of aerosolized medications. Br J Clin Pharmacol.

[45] Shur J, Lee S, Adams W, Lionberger R, Tibbatts J, Price R (2012). Effect of device design on the in vitro performance and comparability for capsule-based dry powder inhalers. AAPS J.

[46] Ulrik CS, Lange P (2011). Targeting small airways in asthma: improvement in clinical benefit?. Clin Respir J.

[47] Burgel PR, Bourdin A, Chanez P, Chabot F, Chaouat A, Chinet T, de Blic J, Devillier P, Deschildre A, Didier A (2011). Update on the roles of distal airways in COPD. Eur Respir Rev.

[48] Burgel PR, de Blic J, Chanez P, Delacourt C, Devillier P, Didier A, Dubus JC, Frachon I, Garcia G, Humbert M (2009). Update on the roles of distal airways in asthma. Eur Respir Rev.

[49] Verkindre C, Fukuchi Y, Flemale A, Takeda A, Overend T, Prasad N, Dolker M (2010). Sustained 24-h efficacy of NVA237, a once-daily long-acting muscarinic antagonist, in COPD patients. Respir Med.

[50] Vogelmeier C, Ramos-Barbon D, Jack D, Piggott S, Owen R, Higgins M, Kramer B (2010). Indacaterol provides 24-h bronchodilation in COPD: a placebo-controlled blinded comparison with tiotropium. Respir Res.

[51] Decramer ML, Hanania NA, Lotvall JO, Yawn BP (2013). The safety of long-acting beta2-agonists in the treatment of stable chronic obstructive pulmonary disease. Int J Chronic Obstruct Pulmonary Dis.

